# Supplementary material for: Association between oral microbiome and seven types of cancers in East Asian population: a two-sample Mendelian randomization analysis
Source: Front Mol Biosci. 2023 Nov 21;10:1327893. doi: 10.3389/fmolb.2023.1327893 (PMC10702768; doi:10.3389/fmolb.2023.1327893)
Supplement: Supplementary file 1 [file DataSheet1.ZIP › Supplementary Materials/MR plots for tongue/tongue═╝/Ovarian cancer/pheno.2361_to_ovarian cancer_forest.pdf]

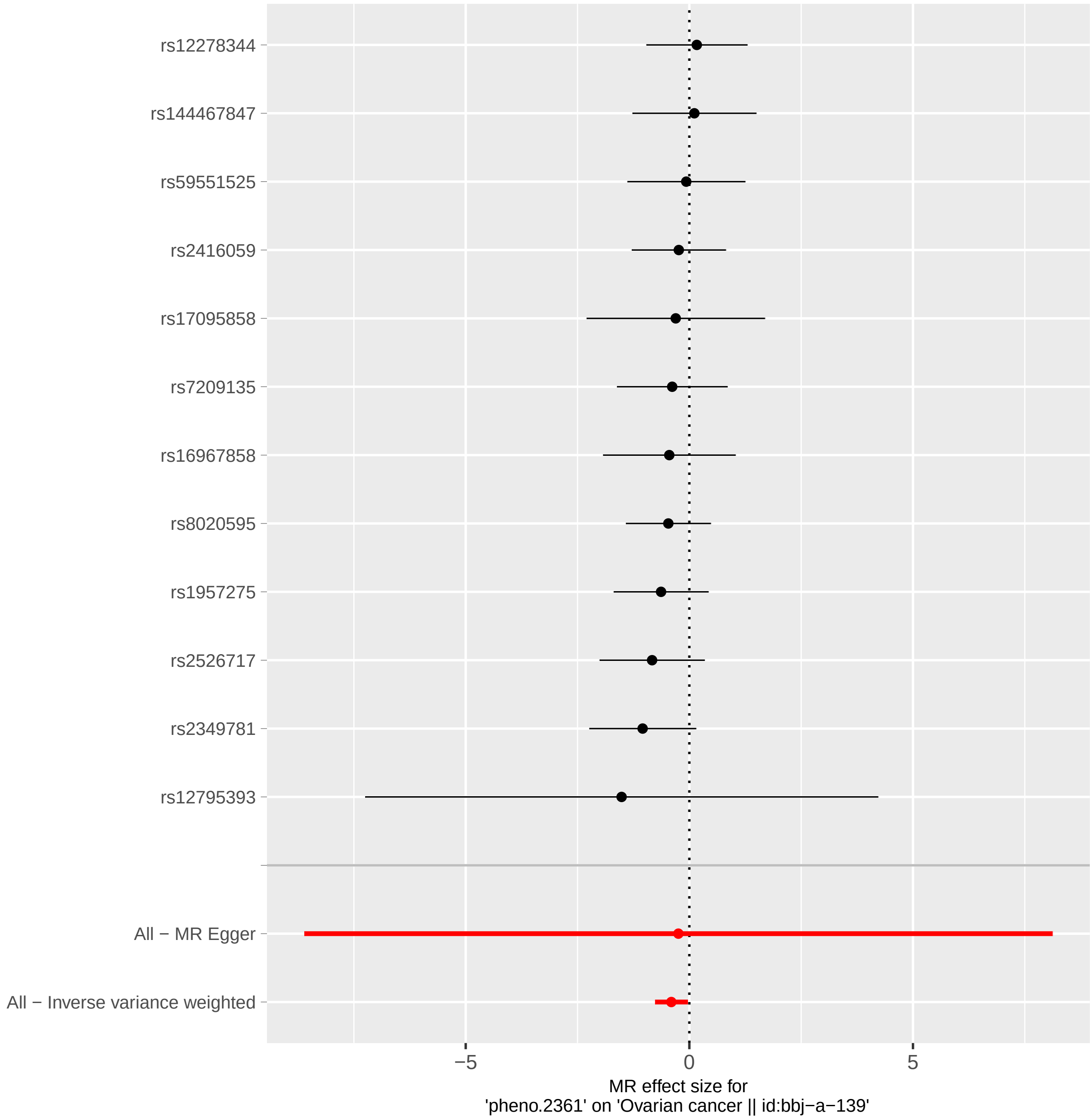
rs12278344
rs144467847
rs59551525
rs2416059
rs17095858
rs7209135
rs16967858
rs8020595
rs1957275
rs2526717
rs2349781
rs12795393
All − MR Egger
All − Inverse variance weighted
−5
0
5
MR effect size for
'pheno.2361' on 'Ovarian cancer || id:bbj−a−139'
